# A Simple Method for the Prediction of Therapeutic Proteins (Monoclonal and Polyclonal Antibodies and Non-Antibody Proteins) for First-in-Pediatric Dose Selection: Application of Salisbury Rule

**DOI:** 10.3390/antib11040066

**Published:** 2022-10-21

**Authors:** Iftekhar Mahmood

**Affiliations:** Mahmood Clinical Pharmacology Consultancy, LLC 1709, Piccard DR, Rockville, MD 20850, USA; iftekharmahmood@aol.com; Tel.: +1-301-838-4555

**Keywords:** pediatric dose, clearance, body weight, Salisbury Rule

## Abstract

In order to conduct a pediatric clinical trial, it is important to optimize pediatric dose as accurately as possible. In this study, a simple weight-based method known as ‘Salisbury Rule’ was used to predict pediatric dose for therapeutic proteins and was then compared with the observed pediatric dose. The observed dose was obtained mainly from the FDA package insert and if dosing information was not available from the FDA package insert then the observed dose was based on the dose given to an age group in a particular study. It was noted that the recommended doses of most of the therapeutic proteins were extrapolated to pediatrics from adult dose based on per kilogram (kg) body weight basis. Since it is widely believed that pediatric dose should be selected based on the pediatric clearance (CL), a CL based pediatric dose was projected from the following equation: Dose in children = Adult dose × (Observed CL in children/Observed adult CL). In this study, this dose was also considered observed pediatric dose for comparison. A ±30% prediction error (predicted vs. observed) was considered acceptable. There were 21 monoclonal antibodies, 5 polyclonal antibodies in children ≥ 2 years of age, 4 polyclonal antibodies in preterm and term neonates, and 11 therapeutic proteins (non-antibodies) in the study. In children < 30 kg body weight, the predicted doses were within 0.5–1.5-fold prediction error for 87% (monoclonal antibody), 100% (polyclonal antibody), and 92% (non-antibodies) observations. In children > 30 kg body weight, the predicted doses were within 0.5–1.5-fold prediction error for 96% (monoclonal antibody), 100% (polyclonal antibody), and 100% (non-antibodies) observations. The Salisbury Rule mimics more to CL-based dose rather than per kg body weight-based extrapolated dose from adults. The Salisbury Rule for the pediatric dose prediction can be used to select first-in-children dose in pediatric clinical trials and may be in clinical settings.

## 1. Introduction

Due to physiological and biochemical differences between children and adults, dosing of drugs in children requires a thorough consideration. Unlike first in adult dose, where the primary focus is the safety (not necessarily efficacy), in children both safety and efficacy are the focus because for ethical reasons children can only be dosed when they need medicine for an underlying disease [1,2].

Pediatric diseases may differ from those of adults in terms of clinical or biological features, mechanisms, etiology, and the course of disease. The pharmacokinetics (PK) and pharmacodynamics (PD) of drugs, in most instances, are different in children than adults [1,2].

In pediatric drug development, the selection of first-in-children dose is very important. Before administering a drug to pediatric population, generally the PK information and a safe and efficacious dose in adult population are known which can be used to select first-in-children dose. Over the years, empirical models such as allometric scaling, modeling and simulation generally using adult data, and physiologically based pharmacokinetic (PBPK) models have been suggested to select the first-in-children dose [2,3,4,5,6].

Several simple pediatric dosing rules have been described in the literature. These rules are: Clark’s rule (2–17 years), Clark’s surface area rule, Young’s rule, Webster’s rule, Fried’s rule, and Shirkey’s BSA recommendation [7,8]. However, these methods were developed for small molecules and the predictive power of these widely known rules are considered unreliable. Munzenberger and McKercher [8] evaluated the performance of several pediatric dosing rules (Clark’s weight rule, Clark’s surface area rule, Young’s age rule, and Shirkey’s dosing recommendations) with the actual doses administered to pediatric patients. The authors’ overall conclusion was that these pediatric dosing rules although, simple but were unreliable.

Clearance (CL) is a very important PK parameter. CL is inverse of exposure (area under the curve (AUC). It is widely believed that pediatric dose should be selected based on the exposure or CL of the drug rather than per kg body weight basis extrapolated from adults [1,2,4]. It was noted (from the FDA package inserts or from the literature) that the recommended doses for the most of the macromolecules used in this study were extrapolated to pediatrics from adult dose based on per kilogram (kg) body weight basis. Therefore, besides an observed dose based on per kg body weight, another observed dose was also chosen based on the observed adult and children CL values (described in the Section 2).

The objective of this study was to evaluate a simple method proposed by Lack and Stuart Known as ‘Salisbury Rule’ [7]. The method was found useful for pediatric dosing for small molecules [7]. The method is based on body weight. The method was originally developed for small molecules and has not been evaluated for macromolecules. Therefore, considering the simplicity of this model, the objective of this study was to evaluate if Salisbury Rule can be used to predict pediatric dose of macromolecules or therapeutic proteins (monoclonal and polyclonal antibodies as well as non-antibodies).

## 2. Methods

From the literature, age, body weight, clearance (CL) and pediatric doses of therapeutic proteins were obtained [9,10,11,12,13,14,15,16,17,18,19,20,21,22,23,24,25,26,27,28,29,30,31,32,33,34,35,36,37,38,39,40,41,42,43,44,45,46,47,48,49,50,51,52,53,54,55,56,57,58,59,60,61,62,63] The recommended pediatric doses of these drugs were obtained from the FDA package insert, and/or from the studies where a particular dose was given to children with certain body weights.

### 2.1. Salisbury Rule

The following method known as ‘Salisbury Rule’ was used for the prediction of dose in children (from preterm neonates (only for some polyclonal antibodies) to young children and adolescents). This method was proposed by Lack and Stuart-Taylor and is as follows [7]:

For pediatric patients weighing less than 30 kg:

2 × weight in kilograms = % of adult dose(1)

For pediatric patients weighing greater than or equal to 30 kg but less than 70 kg:

weight in kilograms + 30 = % of adult dose(2)

Since the observed doses (from FDA PI or literature-based) of the most macromolecules used in this study were extrapolated to pediatrics from adult dose based on per kilogram (kg) body weight basis, CL based pediatric dose was determined. This dose was based on the observed adult and children CL and was also used as the observed dose for comparison purpose.

dose in children = adult dose × (observed CL in children/observed adult CL)(3)

The predicted pediatric dose by Salisbury Rule was compared with the recommended FDA package insert or literature-based dose as well as based on the dose obtained from Equation (3) (CL based).

### 2.2. Statistical Analysis

Percent prediction error between the observed and predicted dose was calculated according to the following equation:

% error = [(predicted-observed)/observed] × 100(4)

The ratio or fold-error between predicted and observed was calculated as follows:

ratio or fold-error = (predicted/observed)(5)

Generally, a 2-fold prediction error is considered acceptable. However, this author considers a 2-fold prediction error too high and of little practical value even for the first-time-pediatric dose selection. Percent prediction error of ≤30% (>0.7 or ≤1.3-fold) on either side of 100% (+ or −) was considered reasonably accurate prediction of the pediatric dose. In this study, prediction error of + >30% (>130% or >1.3-fold) and − >30% (<70% or <0.7-fold) were considered overestimation or underestimation of the observed dose, respectively.

It should be noted that the predicted dose was a single value and was compared with a single observed value. In real life situation, for many drugs, there will be a dose range in a given age group and this may minimize the prediction error noted in this study. Furthermore, for some drugs, in clinical practice, the observed dose used in this study may differ from the recommended dose due to the difference in the response. Considering these facts, a 30% prediction error by Salisbury Rule was considered accurate for the first-in-pediatric dose selection to initiate a clinical trial and possibly in clinical settings.

## 3. Results

**Figure 1:**
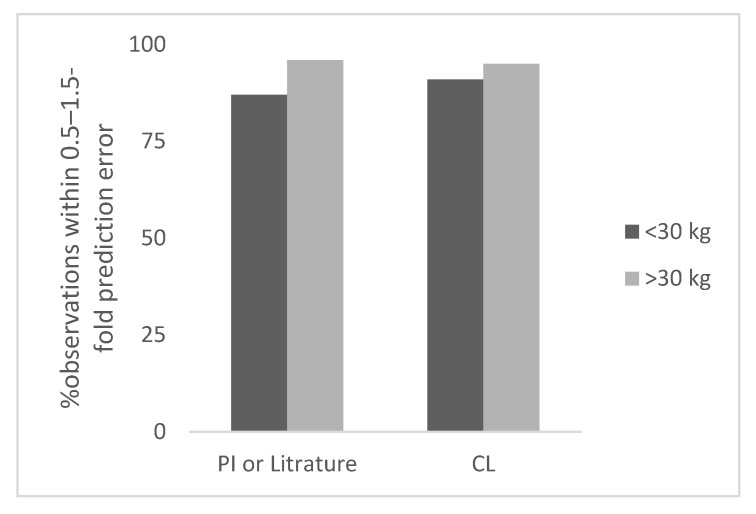
Percent observation within 0.5–1.5-fold prediction error for monoclonal antibodies.

**Figure 2:**
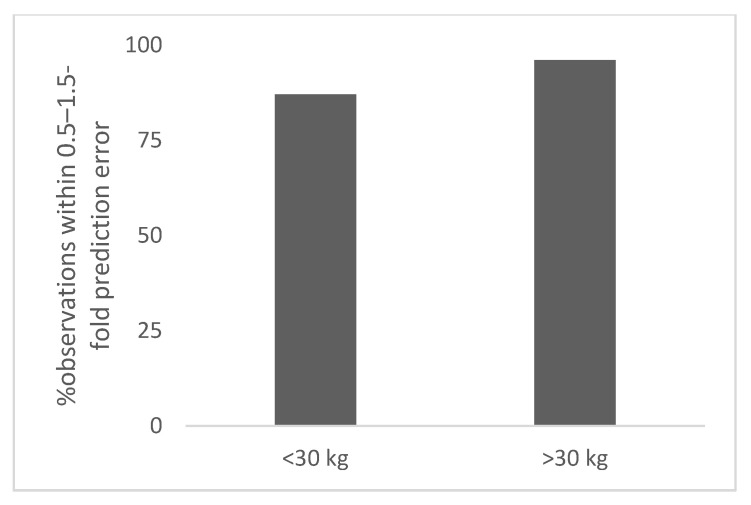
Percent observation within 0.5–1.5-fold prediction error for polyclonal antibodies (>2 years of age).

**Figure 3:**
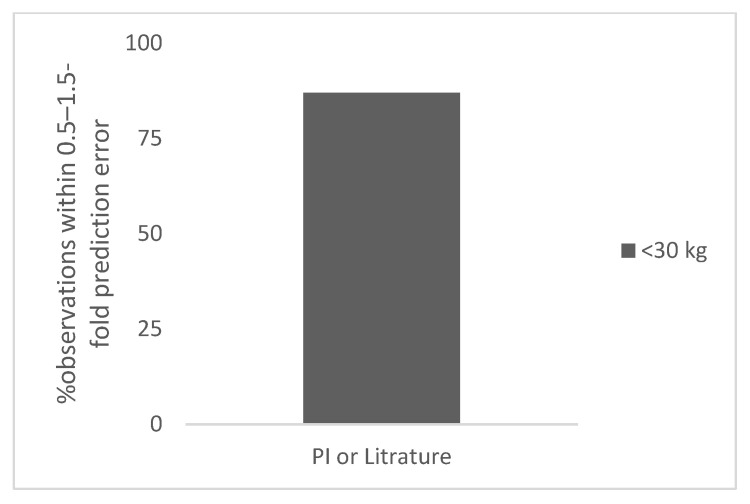
Percent observation within 0.5–1.5-fold prediction error for polyclonal antibodies in the neonates.

**Figure 4:**
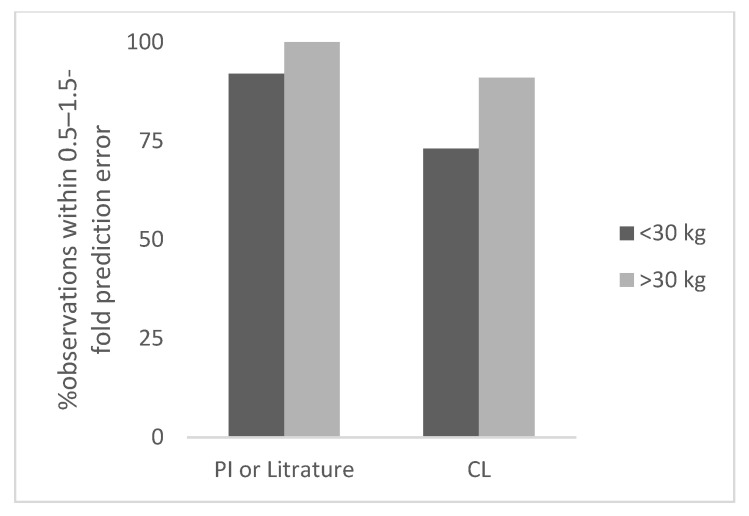
Percent observation within 0.5–1.5-fold prediction error for non-antibodies.

The results of this study are summarized below and in Table 1, Figure 1, Figure 2, Figure 3 and Figure 4, and Supplementary Tables S1–S4.

### 3.1. Monoclonal Antibodies

A total of 21 monoclonal antibodies with different age groups and body weights were analyzed. The body weight of children ranged from 4.5 to 60 kg. The total number of observations was 31 and 28 for <30 kg and ≥30 kg body weight children, respectively. The observed and predicted doses in children for monoclonal antibodies are shown in Table S1.

The prediction error in children for <30 kg ranged from 0% to 60% (the next highest error was 55%). The percent observations within 0.5–1.5-fold, and 0.7–1.3-fold prediction error was 87% and 36%, respectively (Table 1). There were 14 observations for which dose was not based on per kg body weight based extrapolation. Out of these 14 observations, 11 (79%) observations had prediction error ≤30%. (within 0.7–1.3-fold).

For many monoclonal antibodies PK studies in children were not conducted therefore, pediatric clearance values were not available for all 21 monoclonal antibodies analyzed in this study. Therefore, the total number of observations was 21 for <30 kg body weight children. Based on CL based observed dose, the prediction error in children for <30 kg body weight ranged from 0% to 94% (the next highest error was 63%). The percent observations within 0.5–1.5-fold, and 0.7–1.3-fold prediction error was 91% and 67%, respectively (Table 1). In Figure 1, the percent observation within 0.5–1.5-fold prediction error for monoclonal antibodies is shown.

Overall, predicted pediatric dose by Salisbury Rule was more accurate by CL based observed dose than per kg body weight basis dose. When pediatric dose was not based on per kg body weight basis, the pediatric dose prediction was fairly accurate (79% with ≤30% prediction error).

The total number of observations was 28 for ≥30 kg body weight children. The observed and predicted doses in children for monoclonal antibodies are shown in Table S1. The prediction error in children for ≥30 kg ranged from 3% to 44% (the next highest error was 35%). The percent observations within 0.5–1.5-fold and 0.7–1.3-fold prediction error was 96%and 89%, respectively (Table 1).

Based on CL based observed dose (*n* = 21), the prediction error in children for ≥30 kg ranged from 1% to 53% (the next highest error was 36%). The percent observations within 0.5–1.5-fold and 0.7–1.3-fold prediction error was 95% and 86%, respectively (Table 1).

The results indicated that the Salisbury Rule predicted dose much more accurately in children weighing ≥30 kg than the children weighing <30 kg (Table 1). In children <30 kg body weight, the predicted dose on per kg body weight was less accurate than the CL based dose with respect to ≤30% (Table 1). This is not surprising because the pediatric dose prediction by Salisbury Rule was proposed to avoid per kg body weight extrapolation from adults to children. As mentioned earlier, the predicted dose of monoclonal antibodies in children <30 kg substantially improved if the dose was not selected based on per kg body weight basis.

### 3.2. Polyclonal Antibodies (Not Premature or Term Neonates)

A total of 5 polyclonal antibodies with different age groups (not premature or term neonates) and body weights were studied. The body weight of children ranged from 15 to 50 kg. The total number of observations was 9 for <30 kg body weight children. The observed and predicted doses in children for polyclonal antibodies based on per kg extrapolation from adult dose are shown in Table S2. The prediction error in children for <30 kg ranged from 37% to 40% (8 with 40% prediction error and 1 with 37% prediction error). The percent observation within 0.5–1.5-fold and 0.7–1.3-fold prediction error was 100% and 0%, respectively (Table 1).

Dose was not selected based on CL because only slight difference was noted in CL in children from 2 years to 16 years and adults. Therefore, the dose of polyclonal antibodies in children was based on per kg basis extrapolated from adults (original studies).

The total number of observations was 5 for ≥30 kg. The observed and predicted doses in children for polyclonal antibodies based on per kg extrapolation from adult dose are shown in Table S2. The prediction error in children for ≥30 kg ranged from 12% to 14% (5 with 12% prediction error and 1 with 14% prediction error). The percent observation within 0.5–1.5-fold and 0.7–1.3-fold prediction error was 100% (Table 1). In Figure 2, the percent observation within 0.5–1.5-fold prediction error for polyclonal antibodies is shown.

### 3.3. Polyclonal Antibodies (Premature and Term Neonates)

There were four polyclonal antibodies for which data were available for premature (*n* = 6) or term (*n* = 3) neonates. The total number of observations was 9. The body weight of these children ranged from 1 to 3 kg. The prediction error in the neonates ranged from 12% to 124% (the next highest error was 44%). The observed and predicted doses in children for polyclonal antibodies in premature and term children are shown in Table S3. The percent observation within 0.5–1.5-fold and 0.7–1.3-fold prediction error was 89% and 67%, respectively (Table 1). In Figure 3, the percent observation within 0.5–1.5-fold prediction error for polyclonal antibodies in neonates is shown.

The predicted dose of polyclonal antibodies in neonates by Salisbury Rule was more accurate than the older children. The percent error of ≤30% for predicted dose from Salisbury Rule for older children was 0% whereas, for neonates the prediction error was 67%. This was because the dose received by the older children was based on per kg weight basis extrapolated from adults whereas, the neonates received different doses of polyclonal antibodies in different studies. The dose ranged from 250 to 1000 mg/kg and was not extrapolated based on the adult dose on per kg body weight basis. The dose of polyclonal antibodies were selected by the investigators to evaluate the safety and efficacy in the neonates. The lowest dose 250 mg/kg produced the highest prediction error of 124%. It should be recognized that the lowest dose of a polyclonal antibody in preterm neonates (like older children) may range from 400 to 500 mg/kg. The projected dose by Salisbury Rule for the group who received 250 mg/kg in the clinical trial was 560 mg/kg which reconciles very well with the current dosing standard of polyclonal antibodies in children.

For polyclonal antibodies, the clinical experience indicates that dose extrapolated from adults for children 2–16 years is acceptable. The limited PK data in children (2–16 years) indicate that CL of polyclonal antibodies based on per kg body weight is similar to adults. Therefore, dose extrapolation based on per kg body weight from adults is justifiable for polyclonal antibodies.

On the other hand, the limited PK data in premature and term neonates indicate that CL on per kg body weight is higher in neonates than adults and as a result the neonates may need higher doses (per kg body weight) of polyclonal antibodies than adults.

### 3.4. Therapeutic Proteins (Non-Antibodies)

A total of 11 therapeutic proteins with different age groups and body weights were studied. The body weight of children ranged from 4 to 60 kg. The total number of observations was 12 for <30 kg body weight children. The observed and predicted doses in children for therapeutic proteins based on per kg body weight or CL are shown in Table S4. The prediction error in children for <30 kg ranged from 10% to 53% (the next highest error was 41%). The percent observations within 0.5–1.5-fold and 0.7–1.3-fold prediction error was 92% and 42%, respectively.

Based on CL based observed dose (*n* = 11), the prediction error in children for <30 kg ranged from 4% to 75% (the next highest error was 58%). The percent observations within 0.5–1.5-fold and 0.7–1.3-fold prediction error was 73% and 64%, respectively (Table 1). Overall, the prediction of pediatric dose of therapeutic proteins was more accurate by Salisbury Rule when pediatric dose was CL based.

The total number of observations was 11 for ≥30 kg children. The observed and predicted doses in children for therapeutic proteins based on per kg dose or CL are shown in Table S4. The prediction error in children for ≥30 kg ranged from 3% to 44% (the next highest error was 34%). The number of observations within 0.5–1.5-fold and 0.7–1.3-fold prediction error was 100% and 82%, respectively, (Table 1).

Based on CL based observed dose (*n* = 11), the prediction error in children for ≥30 kg ranged from 3% to 69% (the next highest error was 37%). The number of observations within 0.5–1.5-fold and 0.7–1.3-fold prediction error was 91% and 82%, respectively. In Figure 4, the percent observation within 0.5–1.5-fold prediction error for non-antibody proteins is shown.

As observed with monoclonal and polyclonal antibodies, the results for non-antibody proteins also indicated that the Salisbury Rule predicted dose much more accurately in children weighing ≥30 kg than the children weighing <30 kg (Table 1). The prediction of pediatric dose in children <30 kg was more accurate by Salisbury Rule when predicted dose was compared with CL based observed dose.

## 4. Discussion

It is a general practice to extrapolate pediatric dose from adults (especially, in neonates) based on the body weight basis, mainly due to the difficulties in conducting studies in children. This practice although, simple but in many cases may not provide satisfactory outcome related to the safety and efficacy in children. It is widely believed that children should be dosed based on exposure or CL rather than dose extrapolated from adults on per kg body weight. Based on per kg body weight, in general, children have higher clearance than adults hence, dose adjustment is necessary in pediatric population especially, when dose is given on per kg body weight. 

This study is an attempt to develop a simple model proposed by Lack and Stuart known as ‘Salisbury Rule’ [7] to predict the pediatric dose for macromolecules. Although, the method is based on body weight but is not based on a linear system. Salisbury Rule has not been widely used or extensively evaluated and this may be due to the misconception that the method uses body weight. The Rule was proposed for small molecules and its suitability for the pediatric dose prediction was noted for small molecules with limited data [7]. This Rule has not been applied to macromolecules and this study is an attempt to evaluate the predictive power of Salisbury Rule for pediatric dose prediction for macromolecules.

Although several simple models as described previously (Section 1) are available for the selection of pediatric dose (mainly for small molecules), these methods are not considered optimum [8]. It has been shown that if allometry is appropriately applied then allometric scaling provides fairly accurate estimates of CL and dose in pediatrics for both small and large molecules [64,65,66,67].

Although, the acceptable criteria in this study was set to ≤30% prediction error for the first-in-pediatric dosing clinical trial a 40–50% prediction error may be acceptable because a pediatric clinical trial should not revolve around only a single dose. Several appropriate doses should be tested to find an optimum pediatric dose of a drug.

Salisbury Rule, which is based on body weight, performed reasonably well across the two body weight groups. In children <30 kg body weight, in terms of ≤30% prediction error, the prediction was poor for those drugs whose doses were extrapolated from adults based on per kg body weight. This was true for all three classes of macromolecules.

Pediatric doses extrapolated from adults based on per kg body weight were over-predicted by Salisbury Rule in many children <30 kg body weight. There were 17 out of 31 (55%) observations for monoclonal antibodies extrapolated from adults based on per kg body weight and all observations were predicted with >30% prediction error. The prediction error was mostly around 40%. In children <30 kg body weight, for 14 observations, pediatric dose was not extrapolated from adult dose based on body weight and out of these 14 observations, 11 observations (79%) were within ≤30% prediction error (Table 1).

On the other hand, in children ≥30 kg body weight, the prediction error of ≤30% was observed for approximately 90% observations (Table 1). For children ≥30 kg body weight when dose was extrapolated from adults based on per kg body weight, the prediction error was not as high as children <30 kg body weight. Out of 28 observations, only 2 observations (39% and 60%) were predicted with >30% prediction error. There were 25 out of 28 observations (monoclonal antibodies) which were predicted within ≤30% prediction error (Table 1).

When the pediatric dose was predicted from Salisbury Rule and compared with CL based observed dose, a substantial improvement in dose prediction was noted in children <30 kg body weight (Table 1). Sixty-seven percent observations were within ≤30% prediction error for this group (36% vs. 67% for monoclonal antibodies). For children ≥30 kg body weight, the number of observations within ≤30% prediction error was comparable (89% vs. 86% for monoclonal antibodies) whether the pediatric dose was extrapolated from adults based on per kg body weight or CL based dose (Table 1). Similar results were obtained with polyclonal and non-antibodies therapeutic proteins.

For polyclonal antibodies, in children >2 years of age, the dose was extrapolated from adults based on per kg body weight. In children <30 kg body weight (*n* = 9), the error in Salisbury Rule predicted dose was >30% but <50% for all 9 observations. On the other hand, in children >30 kg body weight (*n* = 5), the predicted dose by Salisbury Rule was <30% for all 5 observations.

For polyclonal antibodies, 9 observations were available for the neonates and out of 9 observations, 6 observations (67%) were within ≤30% prediction error. For the neonates, polyclonal doses were not extrapolated from adults based on per kg body weight rather the investigators were trying to determine an optimum dose for safety and efficacy in the neonates and several doses were tested. The data for antibodies in the neonates are scarce and more work is needed to evaluate the predictive performance of Salisbury Rule in the neonates.

The observations noted above clearly indicate that although, Salisbury Rule is based on body weight, the predicted dose is not linear across the body weight and this is the strength of this approach. It is more aligned with the dose predicted based on CL which may be more accurate method for the pediatric dose prediction than extrapolation from per kg body weight from adults. In children <30 kg, the pediatric dose predicted by Salisbury Rule should be considered at least 40% higher if the observed dose is based on per kg body weight extrapolated from adults. This does not mean that the pediatric dose predicted by Salisbury Rule is incorrect because it does not reconcile with the pediatric dose recommended by body weight extrapolation from adults. If one assumes (probably the assumption is correct) that pediatric dose recommendation based on pediatric CL (following a pediatric PK study) provides optimal pediatric dose then the pediatric dose prediction from Salisbury Rule is quite accurate. This is because the Salisbury Rule aligns with CL based dosing rather body weight based dosing. The Rule provides a basis to start a pediatric clinical trial or used in a clinical setting with a very simple approach.

## 5. Conclusions

In this report, a simple method to predict first-in-pediatric dose to initiate a pediatric clinical trial or for the use in clinical settings (when clinical trial-based dose is not available in children) for therapeutic proteins were evaluated. The method was initially proposed by Lack and Stuart for small molecules. The method is weight-based but does not predict pediatric dose linearly (based on per kg body weight). The method recognizes that pediatric dose of a molecule should not be extrapolated from adults based on per kg body weight (a linear system). Based on the results of this study, the predictive power of the method for the prediction of pediatric dose reconciles more with clearance based pediatric dosing rather than per kg body weight extrapolated from adults. The CL based pediatric dosing is widely recognized and considered more accurate than per kg body weight. The Salisbury Rule is simple and can be used on a calculator in a very short period of time. From this study, it seems that weight can be used to predict first-in-pediatric dose to initiate a clinical trial for macromolecules with accuracy. This method may also be used in clinical settings for pediatric dosing. The Salisbury Rule with more data especially, for polyclonal antibodies and in the neonates should be evaluated and implemented due to its accuracy and simplicity.

Complexity does not necessarily provide accuracy over simplicity. In a recent article, Deyme et al. [68] highlighted the usefulness and practical values of simple models. The authors wrote “Conversely, such simple models are the most likely to reach bedside application because of their simplicity. It is critical to balance the pros and cons of each strategy for precision medicine in real-world settings. Models should rather be built in the perspective of future practical application. Indeed, for an efficient in silico-to-bedside transposition, we believe that the more complex is a phenomenon, the simpler should be the mathematical model describing it”.

Considering the Dyme et al.’s realistic and a practical real world views on the complex models and George Box [69] views that “Since all models are wrong the scientist cannot obtain a “correct” one by excessive elaboration”, it is not surprising that in a practical world, simple models are far more attractive and practical than complex models. Research and efforts should be focused on simple models. Simple models are scientifically, commercially, and economically highly desirable (particularly for small drug manufacturing companies) and there is no reason not to use and promote these simple models.

## Figures and Tables

**Table 1:** Prediction of dose based on Salisbury Rule.

Error	Based on FDA Package Insert or Literature		Based on CL	
**Monoclonal (*n* = 21)**	**<30 kg (*n* = 31)**	**≥30 kg (*n* = 28)**	**<30 kg (*n* = 21)**	**≥30 kg (*n* = 21)**
0.5–1.5-fold	27 (87.1)	27 (96.4%)	19 (90.5%)	20 (95.2%)
≤30% (≥0.7–≤1.3)	11 (35.5%)	25 (89.3%)	14 (66.7%)	18 (85.7%)
≥130% (>1.3-fold)	17 (54.8%)	2 (7.1%)	5 (23.8%)	3 (14.3%)
≤70% (≤0.7-fold)	3 (9.7%)	1 (3.6%)	2 (9.5%)	0 (0.0%)
**Polyclonal (*n* = 5)**	**<30 kg (*n* = 9)**	**≥30 kg (*n* = 5)**		
0.5–1.5-fold	9 (100%)	5 (100%)	NA	NA
≤30% (≥0.7–≤1.3)	0 (0%)	5 (100%)	NA	NA
≥130% (>1.3-fold)	9 (100%)	0 (0%)	NA	NA
≤70% (≤0.7-fold)	0 (0%)	0 (0%)	NA	NA
**Polyclonal (premature) (*n* = 4 polyclonal antibodies) *n* = 9 observations, body weight range = 1–3 kg**				
0.5–1.5-fold	8 (89%)	NA	NA	NA
≤30% (≥0.7–≤1.3)	6 (67%)	NA	NA	NA
≥130% (>1.3-fold)	1 (11%)	NA	NA	NA
≤70% (≤0.7-fold)	2 (22%)	NA	NA	NA
**Proteins (non-antibodies) *n* = 11**	**<30 kg (*n* = 12)**	**≥30 kg (*n* = 11)**	**<30 kg (*n* = 11)**	**≥30 kg (*n* = 11)**
0.5–1.5-fold	11 (91.7%)	11 (100%)	8 (72.7%)	10 (90.9%)
≤30% (≥0.7–≤1.3)	5 (41.7%)	9 (81.8%)	7 (63.6%)	9 (81.8%)
≥130% (>1.3-fold)	5 (41.7%)	1 (9.1%)	3 (27.3%)	2 (18.2%)
≤70% (≤0.7-fold)	1 (5%)	1 (9.1%)	2 (18.2)	0 (0%)

## Data Availability

Data is contained within the article or Supplementary Materials.

## Supplementary Materials

The following supporting information can be downloaded at: https://www.mdpi.com/article/10.3390/antib11040066/s1, Table S1: Predicted and observed dose of monoclonal antibodies; Table S2: Predicted and observed dose of polyclonal antibodies; Table S3: Predicted and observed dose of polyclonal antibodies in premature and term neonates; Table S4: Predicted and observed dose of therapeutic protein (non-antibodies).
