# Supplementary material for: A Simple Method for the Prediction of Therapeutic Proteins (Monoclonal and Polyclonal Antibodies and Non-Antibody Proteins) for First-in-Pediatric Dose Selection: Application of Salisbury Rule
Source: Antibodies (Basel). 2022 Oct 21;11(4):66. doi: 10.3390/antib11040066 (PMC9590058; doi:10.3390/antib11040066)
Supplement: Supplementary file 1 [file antibodies-11-00066-s001.zip › antibodies-1882419-supplementary.pdf]

*Supplementary material*

**Table S1.** Predicted and observed dose of monoclonal antibodies.

| Age | BW (kg) | Observed Dose (mg) | | Predicted | Ratio | Ratio |
|---|---|---|---|---|---|---|
| | | PI based | CL based | Salisbury | Salisbury | CL based |
| **Basiliximab, Adult Dose = 20 mg, Pediatric Dose = 12 mg/m², Liver transplantation, Ref [1]** | | | | | | |
| <2 years | 10 (0.29 m²) | 3.5 | 6.9 | 4.0 | 1.15 | 0.58 |
| 3.5 years | 20 (0.79 m²) | 9.5 | 8.7 | 8.0 | 0.84 | 0.92 |
| 8.6 years | 35 (1.2 m²) | 14.4 | 10.2 | 13.0 | 0.90 | 1.28 |
| 13.5 years | 50 (1.4 m²) | 16.8 | 17.8 | 16.0 | 0.95 | 0.90 |
| **Basiliximab, Adult Dose = 20 mg, Pediatric Dose = 12 mg/m², Renal transplantation, Ref [2]** | | | | | | |
| 1-4 years | 25 (0.92 m²) | 11.0 | 8.1 | 10.0 | 0.91 | 1.23 |
| 6-12 years | 40 (1.3 m²) | 15.6 | 10.3 | 16.0 | 1.03 | 1.36 |
| 14-16 years | 55 (1.6 m²) | 19.2 | 16.2 | 17.0 | 0.89 | 1.05 |
| **Bevacizumab, Adult Dose = 10 mg/kg, Pediatric Dose = 10 mg/kg, CNS malignancies, Ref [3,4])** | | | | | | |
| 11 months | 8 | 80 | 158 | 112 | 1.40 | 0.71 |
| 31 months | 16 | 160 | 266 | 224 | 1.40 | 0.84 |
| 25 months | 11 | 110 | 201 | 154 | 1.40 | 0.77 |
| **Infliximab, Adult Dose = 5 mg/kg, Pediatric Dose = 5 mg/kg, Crohn's disease and ulcerative colitis, Ref [5–7]** | | | | | | |
| **>6 years** | 40 | 200 | 280 | 245 | 1.23 | 0.88 |
| **6-17 years** | 45 | 225 | 318 | 263 | 1.17 | 0.83 |
| 0.7-3.1 years | 15 | 75 | 54 | 105 | 1.40 | 1.94 |
| 0.2-6.25 years | 25 | 125 | 121 | 175 | 1.40 | 1.45 |
| **Canakinumab, Adult Dose = 150 mg, Pediatric Dose = 2 mg/kg, if wt >40 kg then fixed dose of 150 mg SJIA, Ref [8–10]** | | | | | | |
| 0.7-1.7 years | 10 | 20 | 29 | 30 | 1.50 | 1.03 |
| 2.7-4.9 years | 25 | 50 | 76 | 75 | 1.50 | 0.98 |
| 5.9-11.1 years | 45 | 150 | 119 | 113 | 0.75 | 0.94 |
| 12-17.2 years | 60 | 150 | 138 | 135 | 0.90 | 0.98 |
| **Canakinumab, Adult Dose = 150 mg, Pediatric Dose = 2 mg/kg, if wt >40 kg then fixed dose of 150 mg CAPS, Ref [8–10]** | | | | | | |
| <2 years | 18 | 36 | 51 | 54 | 1.50 | 1.07 |
| 3.5 years | 24 | 48 | 103 | 72 | 1.50 | 0.70 |
| 8.6 years | 35 | 150 | 141 | 98 | 0.65 | 0.69 |
| 13.5 years | 50 | 150 | 150 | 120 | 0.80 | 0.80 |
| **Daclizumab, Adult Dose = 1 mg/kg, Pediatric Dose = 1 mg/kg, acute organ rejection renal transplant, Ref [11,12]** | | | | | | |
| <5 years | 12 | 12 | 28 | 17 | 1.40 | 0.60 |
| 6-12 years | 29 | 29 | 58 | 41 | 1.40 | 0.70 |
| 13-17 years | 52 | 52 | 78 | 57 | 1.10 | 0.74 |
| **Rilonacept, Adult Dose = 320 mg, Pediatric Dose = 4.4 mg/kg, sJIA, Ref [13])** | | | | | | |
| <6 years | 20 | 88 | 98 | 128 | 1.45 | 1.30 |
| 6-11 years | 40 | 176 | 222 | 224 | 1.27 | 1.01 |
| 12-17 years | 50 | 220 | 320 | 256 | 1.16 | 0.80 |
| **Cetuximab, Adult Dose = 250 mg/m², Pediatric Dose = 250 mg/m², refractory Solid Tumors, Ref [14,15]** | | | | | | |
| 1-12 years | 30 | 300 | 169 | 259 | 0.86 | 1.53 |
| 13-18 years | 55 | 400 | 301 | 367 | 0.92 | 1.22 |
| **Natalizumab, Adult Dose = 300 mg, Pediatric Dose = 3 mg/kg, Crohn's disease, Ref [16,17]** | | | | | | |
| 11-17 years | 50 | 150 | 239 | 240 | 1.60 | 1.01 |
| **Atezolizumab, Adult Dose = 1200 mg, Pediatric Dose = 15 mg/kg, metastatic solid tumors or hematologic malignancies, Ref [18]** | | | | | | |
| <2 years | 9 | 137 | 158 | 158 | 1.38 | 1.60 |
| 2-<12 years | 23 | 338 | 392 | 392 | 1.38 | 1.60 |
| 12-<18 years | 52 | 773 | 896 | 896 | 0.82 | 0.95 |
| **Palivizumab, Adult Dose = 15 mg/kg, Pediatric Dose = 15 mg/kg, respiratory syncytial virus (RSV) , Ref [19,20]** | | | | | | |
| 12.3 months (IM) | 4.5 | 68 | 58 | 95 | 1.39 | 1.63 |
| **Tocilizumab, Adult Dose = 280 mg, Pediatric Dose = <30 kg = 10 mg/kg, >30 kg = 8 mg/kg, PJIA, Ref [21]** | | | | | | |
| 5.2 years | 20 | 200 | 119 | 112 | 0.56 | 0.94 |
| 13.1 years | 50 | 400 | 158 | 224 | 0.56 | 1.21 |
| **Mepolizumab, Adult Dose = 100 mg, Pediatric Dose = <40 kg = 40 mg, ≥40 kg = 100 mg, severe eosinophilic asthma, Ref [22]** | | | | | | |
| 8 years | 27 | 40 | 45 | 54 | 1.35 | 1.20 |
| 10 years | 50 | 100 | 75 | 80 | 0.80 | 1.33 |
| **Urtoxazumab, Adult Dose = 1 mg/kg, Pediatric Dose = 1 mg/kg, hemolytic-uremic syndrome, Ref [23]** | | | | | | |
| 2.9 years | 15 | 15 | 21 | 21 | 1.40 | 1.00 |
| **Abatacept, Adult Dose = 750 mg, Pediatric Dose = 10 mg/kg, JIA, Ref [24]** | | | | | | |
| 6-17 years | 50 | 500 | 731 | 600 | 1.20 | 0.82 |
| **Belatacept, Adult Dose = 5mg/kg, Pediatric Dose = 7.5 mg/kg, kidney transplant, Ref [25,26]** | | | | | | |
| 13-17 years | 59 | 442 | 277 | 312 | 0.70 | 1.12 |

| | | | | | | |
|---|---|---|---|---|---|---|
| **Eculizumab, Adult Dose = 900 mg, Pediatric Dose = 5-<10 kg = 300, 10-<20 kg, 20<30, 30-<40 kg = 600 mg, >40 kg = 900 mg, hemolytic-uremic syndrome, ref [5]** | | | | | | |
| Not known | 8 | 300 | NA | 144 | 0.48 | NA |
| | 15 | 600 | NA | 270 | 0.45 | NA |
| | 25 | 600 | NA | 450 | 0.75 | NA |
| | 35 | 600 | NA | 585 | 0.98 | NA |
| | 50 | 900 | NA | 720 | 0.80 | NA |
| **Adalimumab, Adult Dose = 40 mg, Pediatric Dose = 10-<15 kg = 10 mg, 15-<30 kg = 20 mg, 30-40 kg = 40 mg, JIA or Pediatric Uveitis, Ref [27]** | | | | | | |
| Not known | 12 | 10 | NA | 10 | 0.96 | NA |
| | 25 | 20 | NA | 20 | 1.00 | NA |
| | 40 | 40 | NA | 28 | 0.70 | NA |
| **Ustekinumab, Adult Dose = 45 mg, Pediatric Dose = 0.75 mg/kg for <60 kg, moderate to severe plaque psoriasis, Ref [28]** | | | | | | |
| 12-17 years | 40 | 30 | NA | 32 | 1.05 | NA |
| **Raxibacumab, Adult Dose = 40 mg/kg, Pediatric Dose = 80/kg (<10 kg), 60 mg/kg (>10-40 kg), 40 mg/kg (>40 mg/kg), Inhalational anthrax, Ref [29]** | | | | | | |
| Not known | 10 | 800 | NA | 560 | 0.70 | NA |
| | 25 | 1500 | NA | 1400 | 0.93 | NA |
| | 50 | 2000 | NA | 2240 | 1.12 | NA |
| **Obiltoxaximab, Adult Dose = 16 mg/kg, Pediatric Dose = <15 kg = 32 mg/kg, >15-40 kg = 24 mg/kg, >40 kg = 16 mg/kg, Inhalational anthrax, Ref [30]** | | | | | | |
| Not known | 15 | 480 | NA | 336 | 0.70 | NA |
| | 25 | 600 | NA | 560 | 0.93 | NA |

**Table S2: Predicted and observed dose of polyclonal antibodies.**

| Age | BW (kg) | Observed Dose (mg) | | Predicted | Ratio | Ratio |
|---|---|---|---|---|---|---|
| | | PI based | CL based | Salisbury | Salisbury | CL based |
| **GAMMAPLEX 10% (IV), Adult Dose = 300-800 mg/kg, Pediatric Dose = 300 mg/kg, Ref [31]** | | | | | | |
| 2-5 years | 15 | 4500 | NA | 6300 | 1.40 | NA |
| 6-11 years | 20 | 6000 | NA | 8400 | 1.40 | NA |
| 12-15 years | 50 | 15000 | NA | 16800 | 1.12 | NA |
| **Gamunex-C (IV), Adult Dose = Adult Dose = 300-600 mg/kg, Pediatric Dose = 300 mg/kg, Ref [32]** | | | | | | |
| 2-5 years | 15 | 4500 | NA | 6300 | 1.40 | NA |
| 6-11 years | 20 | 6000 | NA | 8400 | 1.40 | NA |
| 12-15 years | 50 | 15000 | NA | 16800 | 1.12 | NA |
| **Cuvitru (SC), Adult Dose = 220 mg/kg/week, Pediatric Dose = 220 mg/kg/week, Ref [33]** | | | | | | |
| 2-<5 years | 15 | 3300 | NA | 4620 | 1.40 | NA |
| 5-<12 years | 20 | 4400 | NA | 6160 | 1.40 | NA |
| 12-16 years | 50 | 11000 | NA | 12320 | 1.12 | NA |
| **Panzyga (IV), Adult Dose = 300-600 mg/kg, Pediatric Dose = 300 mg/kg, Ref [34]** | | | | | | |
| 2-<6 years | 20 | 6000 | NA | 8400 | 1.40 | NA |
| 6-<12 years | 25 | 7500 | NA | 10500 | 1.40 | NA |
| 12-<16 years | 50 | 15000 | NA | 16800 | 1.12 | NA |
| **Hizentra (SC), Adult Dose = 117 mg/kg, Pediatric Dose = 6-<12 years = 120 mg/kg, 12-<16 years = 115 mg/kg, Ref [35]** | | | | | | |
| 6-<12 years | 25 | 3000 | NA | 4095 | 1.37 | NA |
| 12-<16 years | 50 | 5750 | NA | 6552 | 1.14 | NA |

**Table S3: Predicted and observed dose of polyclonal antibodies in premature and term neonates.**

| BW (kg) | Pediatric Observed Dose (mg/kg) | Pediatric Observed Dose (mg) | Pediatric Predicted Dose (mg) | Ratio |
|---|---|---|---|---|
| **Gammagard (IV), Adult dose = 400-600 mg/kg [36]** | | | | |
| 1.1 | 500 | 550 | 616 | 1.12 |
| 1.1 | 750 | 825 | 616 | 1.12 |
| **Sandoglobulin (IV), Adult dose = 400-600 mg/kg [37]** | | | | |
| 2.6 | 500 | 1300 | 1456 | 1.12 |
| **Gamimune (IV), term, Adult dose = 400 mg/kg (Assumed)* [38]** | | | | |
| 2.87 | 250 | 718 | 1607 | 2.24 |
| 3.04 | 500 | 1520 | 1702 | 1.12 |
| 2.51 | 1000 | 2510 | 1406 | 0.56 |
| **Venoglobulin (IV), Adult dose = 400 mg/kg (Assumed)* [36]** | | | | |
| 1.12 | 1000 | 1120 | 627 | 0.56 |
| 1.06 | 750 | 795 | 594 | 0.75 |
| 1.16 | 500 | 580 | 650 | 1.12 |

*Adult dose was not known for these two polyclonal antibodies therefore, current dosing practice of an initial adult dose of 400 mg was chosen.

**Table S4: Predicted and observed dose of therapeutic protein (non-antibodies).**

| Age | BW (kg) | Observed Dose (mg) | | Predicted | Ratio | Ratio |
|---|---|---|---|---|---|---|
| | | PI based | CL based | Salisbury | Salisbury | CL based |
| **Bivalirudin, Adult Dose = 0.75 mg/kg, Pediatric Dose = 0.75/mg/kg, percutaneous coronary intervention, Ref [39–41]** | | | | | | |
| <30 days | 4 | 3 | 8 | 4 | 1.41 | 0.47 |
| 31 days <2 | 8 | 6 | 17 | 9 | 1.41 | 0.52 |
| 2-<6 | 18 | 13 | 28 | 19 | 1.41 | 0.69 |
| 6-<16 | 41 | 31 | 50 | 38 | 1.23 | 0.76 |
| **Darbepoetin alfa, Adult Dose = 0.5 mcg/kg, Pediatric Dose = 0.5 mcg/kg, chronic kidney disease Ref [42]** | | | | | | |
| 1-17 y | 35 | 18 | 25 | 23 | 1.26 | 0.91 |
| **Darbepoetin alfa, Japanese, Adult Dose = 0.41 mcg/kg, Pediatric Dose = 0.43 & 0.37 mcg/kg, chronic kidney disease Ref [43]** | | | | | | |
| <12 y | 25 | 11 | 16 | 14 | 1.30 | 0.87 |
| >12 y | 40 | 15 | 15 | 20 | 1.34 | 1.34 |
| **Erythropoietin, Adult = 50 U/kg, Pediatric Dose = 50 U/kg, end stage renal failure, Ref [44,45]** | | | | | | |
| 9-12 y | 27 | 1350 | 2625 | 1890 | 1.40 | 0.72 |
| >12-16 y | 41 | 2050 | 3500 | 2485 | 1.21 | 0.71 |
| **Interleukin 11, Adult = 50 mcg/kg, Pediatric Dose = 50 mcg/kg, solid tumors or lymphoma, [46]** | | | | | | |
| >1-3 y | 9 | 433 | 665 | 607 | 1.40 | 0.91 |
| >3-<13 | 26 | 1319 | 1776 | 1847 | 1.40 | 1.04 |
| >13-<17 | 51 | 2532 | 3204 | 2822 | 1.11 | 0.88 |
| **Drotrecogin alfa (activated), Adult Dose = 24 mcg/kg, Pediatric Dose = 24 mcg/kg, chronic kidney disease, Ref [47,48]** | | | | | | |
| <1 year | 6 | 144 | 150 | 202 | 1.40 | 1.34 |
| 1-8 y | 25 | 600 | 531 | 840 | 1.40 | 1.58 |
| >8-18 y | 50 | 1200 | 796 | 1344 | 1.12 | 1.69 |
| **Glulisine, SC, Adult Dose = 0.15 U/kg, Pediatric Dose = 5.9 IU for children, 9.7 IU for adolescents, type 1 diabetes, [49]** | | | | | | |
| 5-11 years | 40 | 6 | 9 | 7 | 1.17 | 0.78 |
| 11-17 years | 64 | 10 | 11 | 10 | 1.0 | 0.91 |
| **Regular Human Insulin (RHI) (SC), Adult Dose = 0.15 U/kg, Pediatric Dose = 5.9 IU for children, 9.7 IU for adolescents, type 1 diabetes, Ref [49,50]** | | | | | | |
| 5-11 years | 40 | 6 | 9 | 7 | 1.17 | 0.78 |
| 11-17 years | 64 | 10 | 11 | 10 | 1.0 | 0.91 |
| **Growth Hormone (LB03002), Adult Dose = 4.7 mg, Pediatric Dose = 0.2 mg/kg, GH deficiency, Ref [51,52]** | | | | | | |
| 7 years | 25 | 5 | 2 | 2 | 0.47 | 1.0 |
| **Enfuvirtide (SC), Adult Dose = 90 mg, Pediatric Dose = 2 mg/kg, HIV-1 Infection, Ref [53,54]** | | | | | | |
| >5-<12 | 27 | 54 | 45 | 49 | 0.90 | 1.07 |
| >12-<17 y | 46 | 92 | 67 | 68 | 0.74 | 1.03 |
| **Peginterferon alpha 2a, Adult Dose = 180 mcg, Pediatric Dose = 104 mcg/m$^2$, Chronic hepatitis C, Ref [55]** | | | | | | |
| 2-8 years | 20 (0.8 m$^2$) | 83 | NA | 72 | 0.87 | NA |

## References


1. Kovarik, J.M.; Gridelli, B.G.; Martin, S.; Rodeck, B.; Melter, M.; Dunn, S.P.; Merion, R.M.; Tzakis, A.G.; Alonso, E.; Bucuvalas, J.; et al. Basiliximab in pediatric liver transplantation: A pharmacokinetic-derived dosing algorithm. *Pediatr. Transplant.* **2002**, *6*, 224–230.
2. Kovarik, J.M.; Offner, G.; Broyer, M.; Niaudet, P.; Loirat, C.; Mentser, M.; Lemire, J.; Crocker, J.F.; Cochat, P.; Clark, G.; et al. A rational dosing algorithm for basiliximab (Simulect) in pediatric renal transplantation based on pharmacokinetic-dynamic evaluations. *Transplantation* **2002**, *74*, 966–971.
3. Lu, J.F.; Bruno, R.; Eppler, S.; Novotny, W.; Lum, B.; Gaudreault, J. Clinical pharmacokinetics of bevacizumab in patients with solid tumors. *Cancer Chemother. Pharm.* **2008**, *62*, 779–786.
4. Gojo, J.; Sauermann, R.; Knaack, U.; Slavc, I.; Peyrl, A. Pharmacokinetics of Bevacizumab in Three Patients Under the Age of 3 Years with CNS Malignancies. *Drugs R D* **2017**, *17*, 469–474.
5. Temrikar, Z.H.; Suryawanshi, S.; Meibohm, B. Pharmacokinetics and Clinical Pharmacology of Monoclonal Antibodies in Pediatric Patients. *Pediatr. Drugs* **2020**, *22*, 199–216.
6. Burns, J.C.; Best, B.M.; Mejias, A.; Mahony, L.; Fixler, D.E.; Jafri, H.S.; Melish, M.E.; Jackson, M.A.; Asmar, B.I.; Lang, D.J.; et al. Infliximab treatment of intravenous immunoglobulin-resistant Kawasaki disease. *J. Pediatr.* **2008**, *153*, 833–838.
7. Shin, D.; Kim, Y.; Kim, Y.S.; Körnicke, T.; Fuhr, R. A Randomized, Phase I Pharmacokinetic Study Comparing SB2 and Infliximab Reference Product (Remicade) in Healthy Subjects. *BioDrugs* **2015**, *29*, 381–388.
8. Sun, H.; Van, L.M.; Floch, D.; Jiang, X.; Klein, U.R.; Abrams, K.; Sunkara, G. Pharmacokinetics and Pharmacodynamics of Canakinumab in Patients With Systemic Juvenile Idiopathic Arthritis. *J. Clin. Pharmacol.* **2016**, *56*, 1516–1527.
9. Kuemmerle-Deschner, J.B.; Ramos, E.; Blank, N.; Roesler, J.; Felix, S.D.; Jung, T.; Stricker, K.; Chakraborty, A.; Tannenbaum, S.; Wright, A.M.; et al. Canakinumab (ACZ885, a fully human IgG1 anti-IL-1β mAb) induces sustained remission in pediatric patients with cryopyrin-associated periodic syndrome (CAPS). *Arthritis Res. Ther.* **2011**, *13*, R34.

10. FDA. Package Insert of ILARIS (Canakinumab) for Cryopyrin-Associated Periodic Syndromes (CAPS); FDA: Silve Spring, MD, USA, 2006.
11. Pescovitz, M.D.; Knechtle, S.; Alexander, S.R.; Colombani, P.; Nevins, T.; Nieforth, K.; Bouw, M.R. Safety and pharmacokinetics of daclizumab in pediatric renal transplant recipients. *Pediatr. Transplant.* **2008**, *12*, 447–455.
12. FDA. Package Insert of ZENAPAX (Daclizumab) for Therapy and Management of Organ Transplant Patients; FDA: Silve Spring, MD, USA, 2003.
13. Autmizguine, J.; Cohen-Wolkowiez, M.; Iiowite, N. Rilonacept Pharmacokinetics in Children with Systemic Juvenile Idiopathic Arthritis. *J. Clin. Pharmacol.* **2015**, *55*, 39–44.
14. Trippett, T.M.; Herzog, C.; Whitlock, J.A.; Wolff, J.; Kuttesch, J.; Bagatell, R.; Hunger, S.P.; Boklan, J.; Smith, A.A.; Arceci, R.J.; et al. Phase I and pharmacokinetic study of cetuximab and irinotecan in children with refractory solid tumors: A study of the pediatric oncology experimental therapeutic investigators' consortium. *J. Clin. Oncol.* **2009**, *27*, 5102–5208.
15. Tan, A.R.; Moore, D.F.; Hidalgo, M.; Doroshow, J.H.; Poplin, E.A.; Goodin, S.; Mauro, D.; Rubin, E.H. Pharmacokinetics of cetuximab after administration of escalating single dosing and weekly fixed dosing in patients with solid tumors. *Clin. Cancer Res.* **2006**, *12*, 6517–6522.
16. Hyams, J.S.; Wilson, D.C.; Thomas, A.; Heuschkel, R.; Mitton, S.; Mitchell, B.; Daniels, R.; A Libonati, M.; Zanker, S.; Kugathasan, S. Natalizumab Therapy for Moderate to Severe Crohn Disease in Adolescents. *J. Pediatr. Gastroenterol. Nutr.* **2007**, *44*, 185–191.
17. FDA. Package Insert of Natalizumab for Adults in Crohn Disease; FDA: Silve Spring, MD, USA, 2004.
18. Shemesh, C.S.; Chanu, P.; Jamsen, K.; Wada, R.; Rossato, G.; Donaldson, F.; Garg, A.; Winter, H.; Ruppel, J.; Wang, X.; et al. Population pharmacokinetics, exposuresafety, and immunogenicity of atezolizumab in pediatric and young adult patients with cancer. *J. Immunother. Cancer* **2019**, *7*, 314.
19. Robbie, G.J.; Zhao, L.; Mondick, J.; Losonsky, G.; Roskos, L.K. Population Pharmacokinetics of Palivizumab, a Humanized Anti-Respiratory Syncytial Virus Monoclonal Antibody, in Adults and Children. *Antimicrob. Agents Chemother*. **2012**, *56*, 4927–4936.
20. FDA. Package Insert of Palivizumab for Children in Respiratory Syncytial Virus (RSV); FDA: Silve Spring, MD, USA, 1998.
21. FDA. Package Insert of Tocilizumab for Polyarticular Juvenile Idiopathic Arthritis (PJIA); FDA: Silve Spring, MD, USA, 2010.
22. Gupta, A.; Pouliquen, I.; Austin, D.; Price, R.G.; Kempsford, R.; Steinfeld, J.; Bradford, E.S.; Yancey, S.W. Subcutaneous mepolizumab in children aged 6 to 11 years with severe eosinophilic asthma. *Pediatr. Pulmonol.* **2019**, *54*, 1957–1967.
23. Lopez, E.L.; Contrini, M.M.; Glatstein, E.; Ayala, S.G.; Santoro, R.; Allende, D.; Ezcurra, G.; Teplitz, E.; Koyama, T.; Matsumoto, Y.; et al. Safety and Pharmacokinetics of Urtoxazumab, a Humanized Monoclonal Antibody, against Shiga-Like Toxin 2 in Healthy Adults and in Pediatric Patients Infected with Shiga-Like Toxin-Producing Escherichia coli. *Antimicrob. Agents Chemother*. **2010**, *54*, 239–243.
24. FDA. Package Insert of Abatacept for Polyarticular Juvenile Idiopathic Arthritis (PJIA); FDA: Silve Spring, MD, USA, 2005.
25. Moudgil, A.; Dharnidharka, V.R.; Feig, D.I.; Warshaw, B.L.; Perera, V.; Murthy, B.; Roberts, M.E.; Polinsky, M.S.; Ettenger, R.B. Phase I study of single-dose pharmacokinetics and pharmacodynamics of belatacept in adolescent kidney transplant recipients. *Am. J. Transplant.* **2018**, *19*, 1218–1223.
26. Shen, J.; Townsend, R.; You, X.; Shen, Y.; Zhan, P.; Zhou, Z.; Geng, D.; Wu, D.; McGirr, N.; Soucek, K.; et al. Pharmacokinetics, Pharmacodynamics, and Immunogenicity of Belatacept in Adult Kidney Transplant Recipients. *Clin. Drug Investig.* **2013**, *34*, 117–126.
27. FDA. Package Insert of Adalimumab for Polyarticular Juvenile Idiopathic Arthritis (PJIA); FDA: Silve Spring, MD, USA, 2002.
28. FDA. Package Insert of STELARA (ustekinumab) for Moderate to Severe Plaque Psoriasis; FDA: Silve Spring, MD, USA, 2009.
29. FDA. Package Insert of Raxibacumab for Inhalational Anthrax; FDA: Silve Spring, MD, USA, 2012.
30. FDA. Package Insert of Obiltoxaximab for Inhalational Anthrax; FDA: Silve Spring, MD, USA, 2016.
31. FDA. *Package Insert of Gamaplex 10%*; FDA: Silve Spring, MD, USA, 2017.
32. FDA. *Package Insert of GAMUNEX-C*; FDA: Silve Spring, MD, USA, 2003.
33. FDA. *Package Insert of Cuvitru*; FDA: Silve Spring, MD, USA, 2016.
34. FDA. *Package Insert of Pangyza*; FDA: Silve Spring, MD, USA, 2018.
35. FDA. *Package Insert of Hizentra*; FDA: Silve Spring, MD, USA, 2010.
36. Noya, F.J.; Rench, M.A.; Garcia-Prats, J.A.; Jones, T.M.; Baker, C.J. Disposition of an immunoglobulin intravenous preparation in very low birth weight neonates. *J. Pediatr.* **1988**, *112*, 278–283.
37. Kyllonen, K.S.; Clapp, D.W.; Kliegman, R.M.; Baley, J.E.; Shenker, N.; Fanaroff, A.A.; Berger, M. Dosage of intravenously administered immune globulin and dosing interval required to maintain target levels of immunoglobulin G in low birth weight infants. *J. Pediatr.* **1989**, *115*, 1013–1016.
38. Weisman, L.E.; Fischer, G.W.; Marinelli, P.; Hemming, V.G.; Pierce, J.R.; Golden, S.M.; Peck, C.C. Pharmacokinetics of Intravenous Immunoglobulin in Neonates. *Vox Sang.* **1989**, *57*, 243–248.
39. Forbes, T.J.; Hijazi, Z.M.; Young, G.; Ringewald, J.M.; Aquino, P.M.; Vincent, R.N.; Qureshi, A.M.; Rome, J.J.; Rhodes, J.F.; Jones, T.K.; et al. Pediatric catheterization laboratory anticoagulation with bivalirudin. *Catheter. Cardiovasc. Interv.* **2011**, *77*, 671–679.
40. Robson, R.; White, H.; Aylward, P.; Frampton, C. Bivalirudin pharmacokinetics and pharmacodynamics: Effect of renal function, dose, and gender. *Clin. Pharmacol. Ther.* **2002**, *71*, 433–439.
41. FDA. Package Insert of ANGIOMAX (Bivalirudin) for Percutaneous Coronary Intervention; FDA: Silve Spring, MD, USA, 2000.

42. Uemura, O.; Hattori, M.; Hataya, H.; Ito, S.; Ito, N.; Akizawa, T. Pharmacokinetics of darbepoetin alfa after single, intravenous or subcutaneous administration in Japanese pediatric patients with chronic kidney disease. *Clin. Exp. Nephrol.* **2014**, *18*, 932–938.
43. Lerner, G.; Kale, A.S.; Warady, B.A.; Jabs, K.; Bunchman, T.E.; Heatherington, A.; Olson, K.; Messer-Mann, L.; Maroni, B.J. Pharmacokinetics of darbepoetin alfa in pediatric patients with chronic kidney disease. *Pediatr. Nephrol.* **2002**, *17*, 933–937.
44. Evans, J.H.; Brocklebank, J.T.; Bowmer, C.J.; Ng, P.C. Pharmacokinetics of recombinant human erythropoietin in children with renal failure. *Nephrol. Dial. Transplant.* **1991**, *6*, 709–714.
45. Kindler, J.; Eckardt, K.U.; Ehmer, B.; Jandeleit, K.; Kurtz, A.; Schreiber, A.; Scigalla, P.; Sieberth, H.G. Single-dose pharmacokinetics of recombinant human erythropoietin in patients with various degrees of renal failure. *Nephrol. Dial. Transpl.* **1989**, *4*, 345–349.
46. Cairo, M.S.; Davenport, V.; Bessmertny, O.; Goldman, S.C.; Berg, S.L.; Kreissman, S.G.; Laver, J.; Shen, V.; Secola, R.; Van De Ven, C.; et al. Phase I/II dose escalation study of recombinant human interleukin-11 following ifosfamide, carboplatin and etoposide in children, adolescents and young adults with solid tumours or lymphoma: a clinical, haematological and biological study. *Br. J. Haematol.* **2005**, *128*, 49–58.
47. Barton, P.; Kalil, A.C.; Nadel, S.; Goldstein, B.; Okhuysen-Cawley, R.; Brilli, R.J.; Takano, J.S.; Martin, L.D.; Quint, P.; Yeh, T.S.; et al. Safety, pharmacokinetics, and pharmacodynamics of drotrecogin alfa (activated) in children with severe sepsis. *Pediatrics* **2004**, *113*, 7–17.
48. FDA. Package Insert of Drotrecogin Alfa (Activated) for Sepsis; FDA: Silve Spring, MD, USA, 2004.
49. Danne, T.; Becker, R.H.A.; Heise, T.; Bittner, C.; Frick, A.D.; Rave, K. Pharmacokinetics, Prandial Glucose Control, and Safety of Insulin Glulisine in Children and Adolescents With Type 1 Diabetes. *Diabetes Care* **2005**, *28*, 2100–2105.
50. Rave, K.; Klein, O.; Frick, A.D.; Becker, R.H. Advantage of Premeal-Injected Insulin Glulisine Compared With Regular Human Insulin in Subjects With Type 1 Diabetes. *Diabetes Care* **2006**, *29*, 1812–1817.
51. Peter, F.; Savoy, C.; Ji, H.-J.; Juhasz, M.; Bidlingmaier, M.; Saenger, P. Pharmacokinetic and pharmacodynamic profile of a new sustained-release GH formulation, LB03002, in children with GH deficiency. *Eur. J. Endocrinol.* **2009**, *160*, 349–355.
52. Bidlingmaier, M.; Kim, J.; Savoy, C.; Kim, M.J.; Ebrecht, N.; de la Motte, S.; Strasburger, C.J. Comparative Pharmacokinetics and Pharmacodynamics of a New Sustained-Release Growth Hormone (GH), LB03002,*Versus*Daily GH in Adults with GH Deficiency. *J. Clin. Endocrinol. Metab.* **2006**, *91*, 2926–2930.
53. Zhang, X.; Lin, T.; Bertasso, A.; Evans, C.; Dorr, A.; Kolis, S.J.; Salgo, M.; Patel, I. Population Pharmacokinetics of Enfuvirtide in HIV-1-Infected Pediatric Patients Over 48 Weeks of Treatment. *J. Clin. Pharmacol.* **2007**, *47*, 510–517.
54. FDA. Package Insert of FUZEON (Enfuvirtide) for HIV-1 Infection; FDA: Silve Spring, MD, USA, 2003
55. Schwarz, K.B.; Mohan, P.; Narkewicz, M.R.; Molleston, J.P.; Nash, S.R.; Hu, S.; Wang, K.; Gries, J.M. Safety, Efficacy and Pharmacokinetics of Peginterferon alpha2a (40 kd) in Children With Chronic Hepatitis C. *J. Pediatr. Gastroenterol. Nutr.* **2006**, *43*, 499–505.
